# Selective elimination of long INterspersed element-1 expressing tumour cells by targeted expression of the HSV-TK suicide gene

**DOI:** 10.18632/oncotarget.16013

**Published:** 2017-03-08

**Authors:** Mariam Chendeb, Robert Schneider, Irwin Davidson, Anas Fadloun

**Affiliations:** ^1^ Department of Functional Genomics and Cancer, Institut de Génétique et de Biologie Moléculaire et Cellulaire, CNRS/INSERM/ULP, Illkirch, Cédex, France; ^2^ Institute of Functional Epigenetics, Helmholtz Center Munich, Neuherberg, Germany; ^3^ Equipe Labellisée of the Ligue Nationale Contre le Cancer, Corvisart, Paris, France

**Keywords:** L1 retrotransposon, Alu element, HSV-TK suicide gene, genome integration, cancer therapy

## Abstract

In gene therapy, effective and selective suicide gene expression is crucial. We exploited the endogenous Long INterspersed Element-1 (L1) machinery often reactivated in human cancers to integrate the Herpes Simplex Virus Thymidine Kinase (HSV-TK) suicide gene selectively into the genome of cancer cells. We developed a plasmid-based system directing HSV-TK expression only when reverse transcribed and integrated in the host genome via the endogenous L1 ORF1/2 proteins and an Alu element. Delivery of these new constructs into cells followed by Ganciclovir (GCV) treatment selectively induced mortality of L1 ORF1/2 protein expressing cancer cells, but had no effect on primary cells that do not express L1 ORF1/2. This novel strategy for selective targeting of tumour cells provides high tolerability as the HSV-TK gene cannot be expressed without reverse transcription and integration, and high selectivity as these processes take place only in cancer cells expressing high levels of functional L1 ORF1/2.

## INTRODUCTION

Suicide gene therapy for cancer treatment, also referred to as gene-directed enzyme pro-drug therapy (GDEPT), aims at selectively targeting cancer cells as an alternative or complementary adjuvant to classical chemotherapies [1]. GDEPT is based on introducing into tumour cells a viral or a bacterial “suicide” gene encoding an enzyme able to activate a non- or mildly toxic pro-drug leading to tumour cell death. The suicide gene has to be selectively active in tumour cells and the pro-drug is delivered either by local or systemic administration and is only activated if the cells express the suicide gene. The most extensively studied suicide gene system is the combination of Herpes Simplex Virus Thymidine Kinase (HSV-TK) and the pro-drug Ganciclovir (GCV). HSV-TK has a high affinity for GCV and catalyses GCV mono-phosphorylation that is further converted into the di- and triphosphate derivatives by cellular kinases. DNA polymerase then incorporates GCV-triphosphate into replicating DNA leading to cell death by polymerase inhibition [2]. In a clinical setting, HSV-TK and GCV were first used to treat ovarian cancer and central nervous system malignancies via in situ transduction (for review see [3]). To be effective GDEPT approaches have to specifically target tumour cells.

The Long Interspersed Element-1 (LINE-1 or L1) retrotransposons make up 17% of the human genome [4]. The vast majority of L1s (approximately 500,000 L1 copies) are no longer mobile due to rearrangements, point mutations, or 5′-truncations [5]. Only around 100 members of the L1-Ta and pre-Ta subfamilies remain transposition-competent [6, 7] and are responsible for the bulk of on-going retro-transposition in humans (reviewed in [8–10]). L1 mobilization primarily occurs via target primed reverse transcription (TPRT), a process catalysed in *cis* by two proteins, ORF1p and ORF2p, translated from the bi-cistronic 6 kb L1 mRNA. The L1 ORF2p comprises endonuclease (EN) and reverse transcriptase (RT) activities essential for L1 retro-transposition and are also responsible for trans-mobilization of Alu and SVA retro-transposons [11–13]. Because of the potential harmful impact of L1 element mobility on genome integrity, their expression is held in check through a variety of genome defence mechanisms [14, 15] for reviews [16, 17]. However, L1 RNA expression has been shown in several adult tissues [18]. In contrast, expression of L1 ORF1/2 protein is not found in normal adult somatic cells, but is seen in many human cancers, including breast cancer [19], human bladder carcinoma, colon carcinoma, melanoma, and fibrosarcoma [20] that exhibit high levels of both L1 RNA and ORF1 protein [8, 9, 21–28]. Similarly, somatic insertions of L1 elements have been described in many cancers indicating that the expressed ORF machinery is functional. There exists little evidence for the presence of functional L1 ORF machinery in normal somatic cells, with only one report of weak ORF1 expression in normal esophagus and where somatic L1 insertions may take place early in the development of Barrett's esopahgus disease [29].

Alu elements are the most abundant Short INterspersed Elements (SINEs), with over one million copies in the human genome [30]. Alu repeats compose greater than 10 % of the mass of the human genome. Full-length Alu elements are approximately 300 bp in length [4, 31]. Alu elements have no open reading frames, but use L1 ORF1p and ORF2p, for their mobility [11, 32]. Of the multiple Alu subfamilies, almost all of the recently integrated Alu elements within the human genome belong to one of several closely related “young” Alu subfamilies: Y, Yc1, Yc2, Ya5, Ya5a2, Ya8, Yb8, and Yb9 with the majority being Ya5 and Yb8 subfamily members [33–36].

It has been shown that endogenously expressed L1 ORF1/2p machinery can support exogenously expressed Alu retroposition [18, 37]. Here we take advantage of the selective expression of L1 ORF1/2 in many cancer cells to specifically express the HSV-TK suicide gene using an expression construct whose genomic integration is mediated by an Alu element. Treatment of HSV-TK-expressing cells with GCV efficiently blocks tumour cell proliferation and spheroid growth. Here we describe for the first time a strategy based on the tumour-specific L1 ORF1/2 expression as means of integrating a suicide gene and eliminating cancer cells that represents a new complement in the treatment of cancer.

## RESULTS

### Designing and optimising a plasmid to integrate and express HSV-TK selectively in L1 ORF1/2 expressing cells using an Alu element

**Figure 1:**
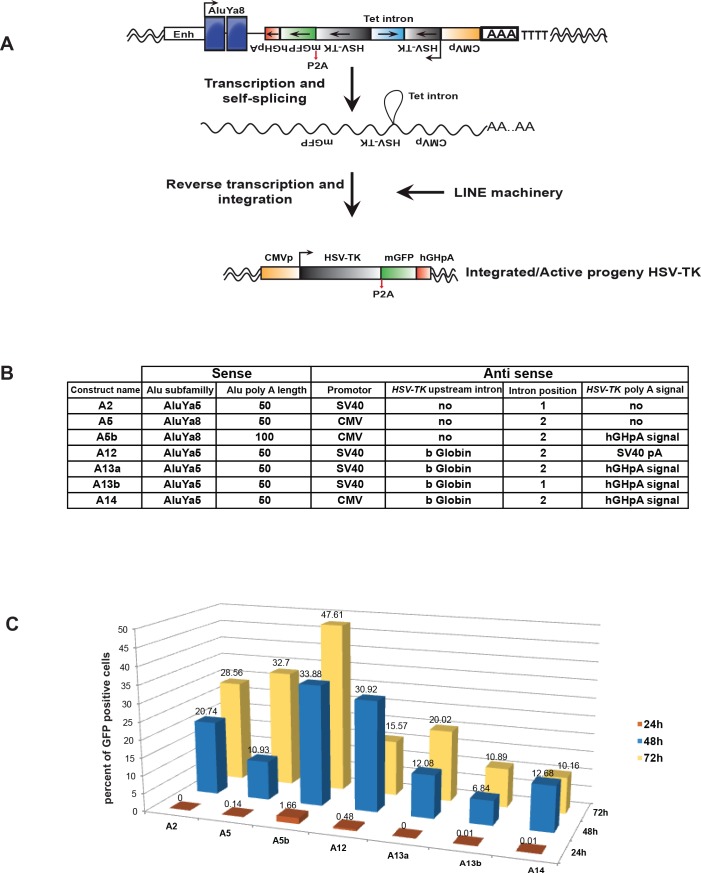
Design and optimisation of vectors **A**. Schematic representation of vector design showing the AluYa8 element flanked with the HSV-TK gene (grey boxes) followed by a pA2 cleavage site and GFP (green box). Both HSV-TK and GFP are on the negative strand with the CMV promoter (yellow box). The HSV-TK coding sequence is interrupted by the presence of an autocatalytic *Tetrahymena* (Tet) intron (light blue box), which can be spliced out of the transposition RNA intermediate (middle). The predicted structure of the resulting *de novo* Alu insertion following splicing and reverse transcription is shown (bottom). **B**. Table summarizing the differences between the constructs. hGHpa is human growth hormone poly A. **C**. Histograms showing the percentage of DAPI stained cells with GFP signal for each construct. 5×10^4^ HEK293T/17 cells were grown in a 12-well plate and transfected with 1μg DNA using FuGENE^®^ (Promega) (DNA/FuGENE ratio 1/6). GFP signal was measured at 24, 48 and 72 hours post transfection using CellInsight, (Cellomics, Thermofisher) and the data were analysed using HCS Studio.

In order to establish a novel plasmid system to express a suicide gene selectively in cancer cells we designed a vector that is derived from a reporter plasmid used to detect Alu retro-transposition with the *neoTet* reporter gene that becomes functional only after a cycle of transcription, reverse transcription and integration (kindly provided by Dr T Heidmann [11]). The *neoTet* reporter gene with its own promoter is encoded on the negative strand and is rendered inactive by the presence of an autocatalytic *Tetrahymena (Tet)* intron that has to be spliced out of the transcribed RNA. This Tetrahymena group I intron can auto-splice and is thus independent from the spliceosome pathway [38]. We replaced the neomycin selection cassette by the HSV-TK gene that was human codon optimized and fused to bright monomer GFP [39] to monitor its expression (Figure 1A). Several different constructs were made to obtain maximal efficiency (Figure 1B). Two different “young” Alu elements, AluYa5 or AluYa8 [33] with internal Pol III promoters were inserted downstream of the enhancer of the Pol III-transcribed Alu-like 7SL RNA gene used in the original plasmid. As the efficiency of Tet self-splicing is dependent on the 5′ splice site delineated by the P1 helix formed by base pairing between the internal guide sequence (IGS) of the intron and the last six nucleotides of the 5′ exon [38], we selected two different positions (1 and 2) to introduce the Tet intron into the HSV-TK gene. These two positions differ by the HSV-TK-IGS pairing at P1 and P10 helixes (Supplemental Figure 1). We also optimised the poly(A) tail length since it has an essential role in L1 and Alu retro-transposition [37, 40–43]. Accordingly, we tried two poly(A) tails of 50 and 100 bases having the RNA polymerase III (Pol III) terminator signal very close in order to maximize activity [37]. To increase Pol II polymerase transcription of the integrated HSV-TK, we compared the CMV and SV40 promoters and we introduced an intron with splice donor and acceptors sites (Figure 1B).

Upon transfection of this vector, the insert is transcribed by Pol III to generate a transcript where the Tet intron is auto-spliced, the transcript is reverse transcribed and then reintegrated into the host genome via the action of the L1 ORF1/2 machinery and the Alu sequence after which Pol II generates a transcript encoding both HSV-TK and GFP (Figure 1A).

In total seven different vectors were generated and their efficiency was evaluated by transfection into HEK293-T cells that express the L1 ORF1p (see below), followed by measurement of the GFP signal. Quantification of the number of GFP expressing transfected cells showed that construct A5b comprising the AluYa8 element, the CMV promoter and a 100 base poly(A) tail was the most efficient (Figure 1C).

### Selective targeting of L1 ORF 1/2 expressing cancer cells

**Figure 2:**
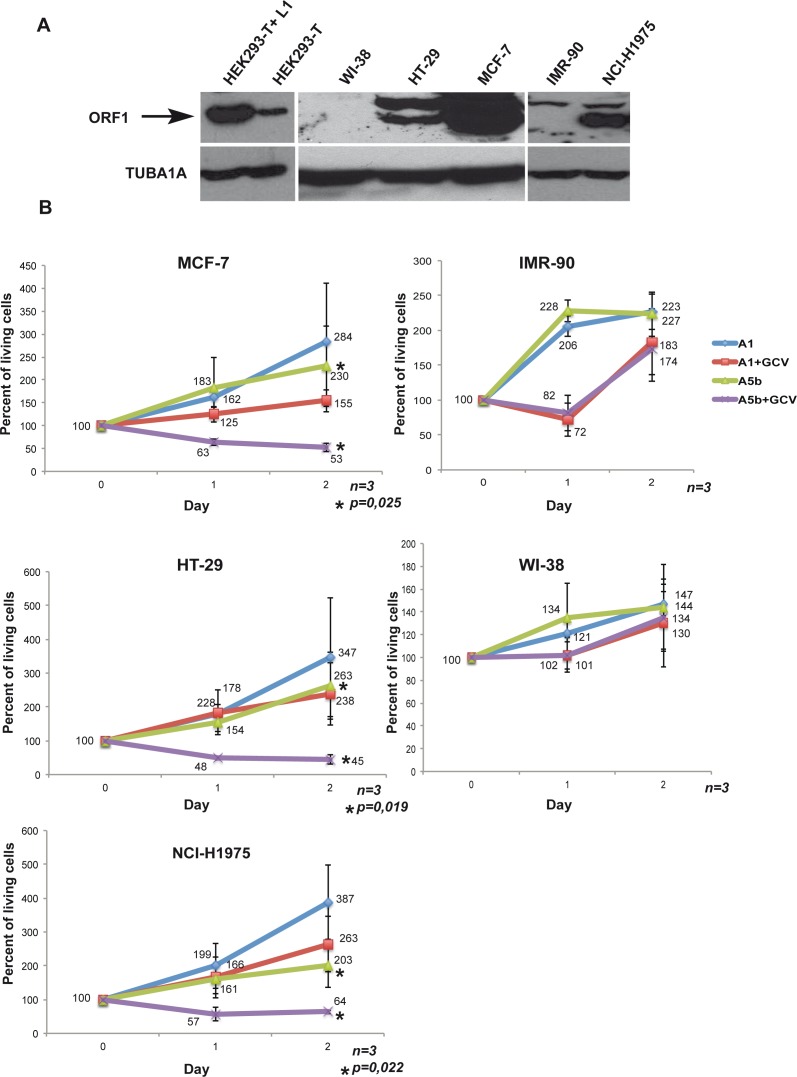
Selective targeting the growth of L1 ORF1/2 expressing cancer cells **A**. Immunoblots showing recombinant ORF1p expression in HEK293-T cells transfected with an L1 expression vector and endogenous ORF1 protein in HEK293-T cells, primary WI-38 and IMR-90 fibroblasts and the HT-29, MCF-7 and NCI-H1975 tumour cells. TUBA1A is used as a loading control. **B**. All indicated cell lines were seeded into a 6-well plate transfected with 4 μg per dish of the A5b or A1 vectors. Transfection was carried out using Lipofectamine 2000^®^ (DNA/lipofectamine 1/3) for HT-29 and FuGENE^®^ (DNA/FuGENE 1/6) for the other lines. After 48 hours, cells were re-transfected and 24 hours later treated with GCV (Sigma-Aldrich) at 10 μg/ml. Living cells were counted at the time of GCV addition (Day 0 =100%), and then at day 1 and day 2. All experiments were performed in triplicate and the data are expressed as mean ± s.d. with a *p*value determined by Student's t-test.

We first screened several tumour cell lines for expression of L1 ORF1/2. Immunoblots with a novel ORF1p antibody showed potent ORF1p expression in HEK293-T cells, MCF-7 breast cancer cells, NCI-H1975 non-small cell lung cancer cells, and HT-29 colorectal adenocarcinoma cells (Figure 2A, note that no reliable and accessible ORF2p antibodies exist so its expression could not be tested in this way). In contrast, no ORF1p was detected in primary IMR-90 and WI-38 non-transformed lung fibroblasts that were used as negative control lines. As an additional control, we transfected a construct (A1) containing a Pol III termination signal within the HSV-TK gene resulting in a shortened transcript that encodes neither an intact HSV-TK, nor a poly(A) signal.

We transfected these cells twice within a 48-hour interval with the A1 and A5b constructs, added GCV 12 hours after the second transfection and counted the living cells immediately (as the base point (Day 0) as well as after 24 and 48 hours. In control A1 transfected MCF-7 cells, GCV addition slowed cell growth such that the number of cells increased only by 50% relative to day 0, whereas they increased by more than 250% in absence of GCV showing a previously reported low level of non-specific toxicity of the drug [44] (Figure 2B). In contrast, GCV treatment of the A5b-transfected cells led to a potent reduction in cell numbers to less than 50% of the starting population. Similar potent reductions in the numbers of viable cells were seen in A5b transfected and GCV treated HT-29 and NCI-H1975 cells. Importantly, no comparable reduction in cell numbers was seen using the IMR-90 and WI-38 cells that did not express L1 ORF1p (Figure 2A and 2B). These data demonstrate that transfection with A5b and GCV treatment can selectively eliminate L1 ORF1p-expressing tumour cells.

**Figure 3:**
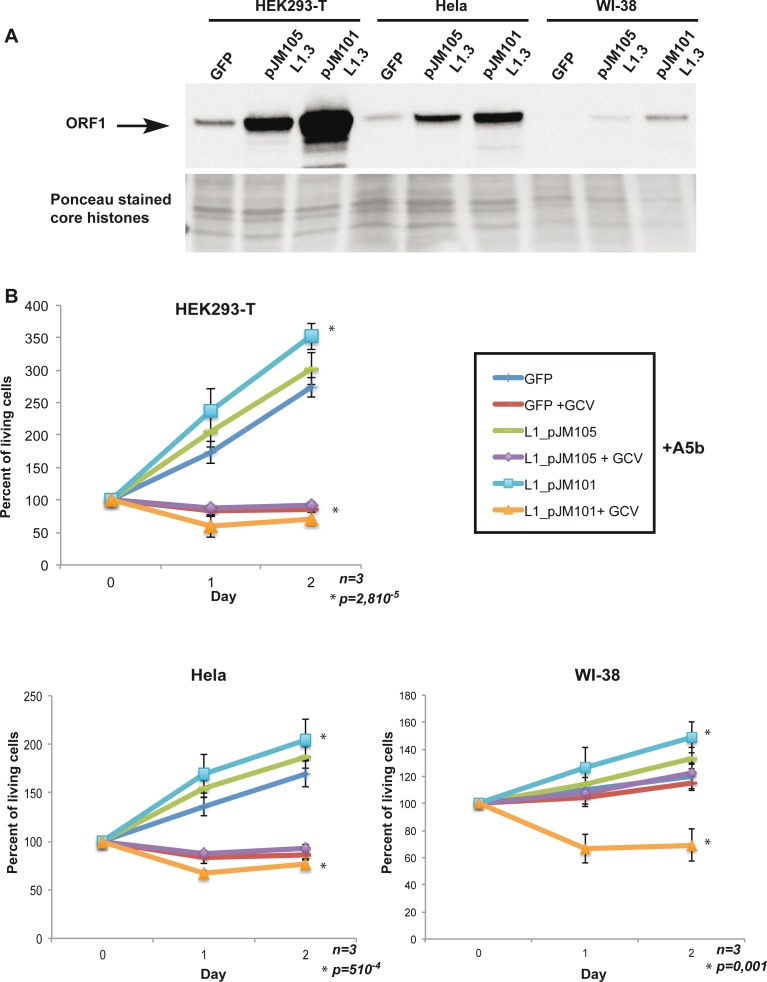
Expression of exogenous wild-type L1 ORF1/2 is necessary and sufficient to confer sensitivity to GCV **A**. Immunoblots showing recombinant ORF1p expression in HEK293-T, HeLa and primary WI-38 cells transfected with a control GFP expression vector or expression vector expressing wild-type ORF1/2p (pJM101L1.3) or wild type ORF1p and a mutated version of ORF2p (D702A) (pJM105L1.3) [45]. Ponceau staining is used as a loading control. **B**. All indicated cell lines were seeded into a 24-well plate transfected with 0.8 μg per well of A5b vector, 24 hours later cells were transfected by triplicate with 0.8 μg/well of GFP, pJM105 and pJM101vectors, 24 hours later GCV (Sigma-Aldrich) were added at 10 μg/ml. Transfection was carried out using FuGENE^®^ (Promega) (DNA/FuGENE 1/6) for HEK293-T cells, X-tremeGENE9^®^ (Sigma-Aldrich) (DNA/X-tremeGENE9 1/4) and Lipofectamine 3000^®^ (Invitrogen) (DNA/lipofectamine 1/3) for WI-38. Living cells were counted as described above. All experiments were performed in triplicate and the data are expressed as mean ± s.d. with a *p*value determined by Student's t-test.

To demonstrate that HSV-TK integration and subsequent GCV-induced cell death were really mediated by the enzymatic activities encoded by the L1 ORF1/2 proteins and thus that L1 expression was the major determinant of sensitivity to our suicide gene approach, we expressed exogenous wild-type ORF1/2 or a version mutated in the reverse transcription (RT) function of ORF2 (D702A) [45] in transformed cells that expressed endogenous L1 ORF1/2 or primary WI-38 cells that did not express endogenous ORF1/2. These constructs or a GFP vector as a negative control were transfected in HeLa or HEK293T transformed cells that we chose as they expressed only low endogenous levels of ORF1 and in primary WI-38 fibroblasts that do not express ORF1 (Figure 3A). After transfection, strong expression of ORF1 was seen in the HeLa or HEK293T cells, while it accumulated to lower, but readily detectable levels in WI-38 cells (Figure 3A). Notably the mutant protein accumulated to lower levels than wild-type in each case.

Cells were co-transfected with these vectors together with the A5b vector with or without GCV treatment. In the absence of any exogenous L1 ORF1/2 the HeLa and HEK293T cells were specifically sensitive to GCV as seen above with the other transformed L1-expressing cells (Figure 3B). Expression of wild-type, but not mutant ORF1/2 led to a mild increase in the proliferation of these cells and also in the sensitivity to GCV treatment such that the overall effect was stronger than with the GFP or mutant ORF1/2 transfected cells (Figure 3B). Strikingly, expression of wild-type ORF1/2 sensitised the WI-38 cells to the effect of GCV. These cells that were normally insensitive to GCV after A5b transfection became sensitive in the presence of wild-type but not mutant ORF1/2.

These data show that expression of exogenous wild-type, but not mutant ORF1/2, in transformed cells expressing endogenous ORF1/2 enhanced their sensitivity to GCV treatment after transfection with the A5b vector. More importantly, expression of wild-type, but not mutant L1 ORF1/2, in WI-38 cells conferred sensitivity to GCV after A5b transfection. Thus, we show that expression of wild-type L1 ORF1/2 is necessary and sufficient to render primary cells sensitive to targeting by the A5b vector and that sensitivity required the L1 encoded RT activity.

Growth of cancer cells as 3D-spheroids can be used to assess their tumour initiating capacity (for reviews see [46, 47]). To test the ability of our strategy to block spheroid growth, cells were transfected as described above as monolayers before culture as 3D spheroids. Alternatively, we introduced the A5b and A1 constructs into Adeno-Associated Virus vectors (AAVs) that were then used to infect cells seeded as monolayers before culture as 3D spheres. Cells were subsequently cultured under non-adherent conditions in presence or absence of GCV and their growth was observed over time. The NCI-H1975, WI-38 and IMR-90 cells did not readily form 3D spheroids in these experiments and these experiments were therefore restricted to the MCF-7 and HT-29 cell lines with the appropriate A1 transfected/infected cells as negative controls.

**Figure 4:**
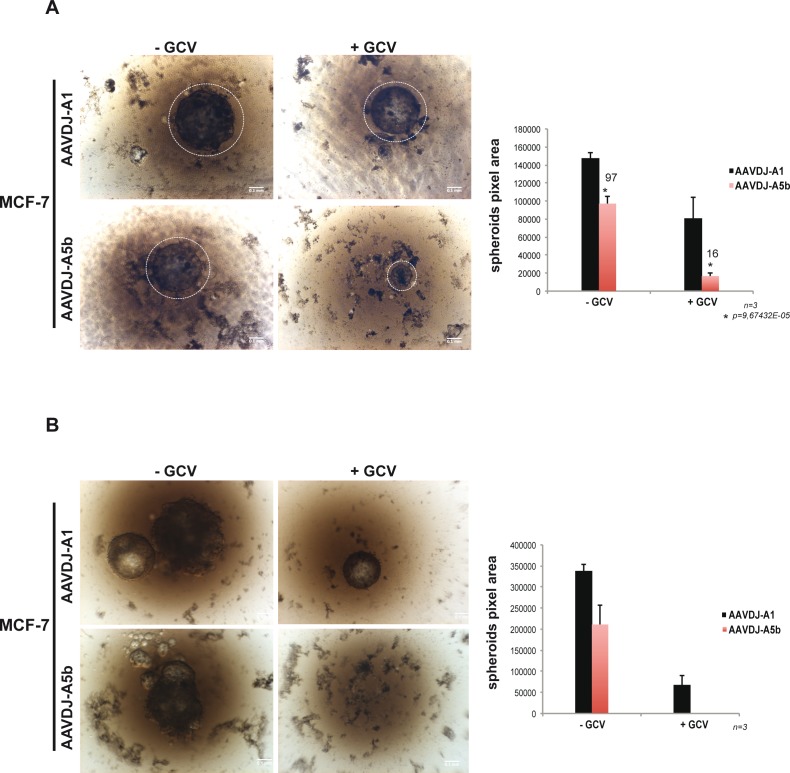
Targeting spheroid growth of MCF-7 cells **A**.-**B**. MCF-7 cells were infected with recombinant A5b and A1-containing AAVs. The A5b and A1 inserts were cloned in pAAVDJ (Cell Biolabs). After two weeks of GCV treatment and double infection (see materiel and methods), spheroid growth was analysed using a bright field Macroscope (Leica M420) (Panel A). The pixel area (right) of each spheroid was determined using Image j (Rasband, W.S., ImageJ, U. S. National Institutes of Health, Bethesda, Maryland, USA, http://imagej.nih.gov/ij/, 1997-2016). Fresh medium was added 1 week after reinfection and spheroids were grown for a further 7 days and imaged a second time (Panel B). All experiments were performed in triplicate and the data are expressed as mean ± s.d. with a *p*value determined by Student's t-test. Scale bars. 100 μm.

MCF-7 spheroids were grown in agarose-coated 96-well plates under conditions where a single spheroid grows in each well. In the absence of GCV, A5b or A1 infected MCF-7 cells formed large spheroids that grew from the agarose surface. GCV treatment of the A1 infected cells led to a reduction in spheroid size, whereas treatment of the A5b infected cells led to a potent inhibition of spheroid growth by 14 days (Figure 4A). Spheroids were re-infected with the AAVs and allowed to grow for a further 14 days. Large spheroids persisted in control A1 infected cells with or without GCV treatment and in the A5b infected cells in absence of GCV. In contrast, spheroid growth was completely abolished in the GCV-treated A5b infected cells (Figure 4B).

**Figure 5:**
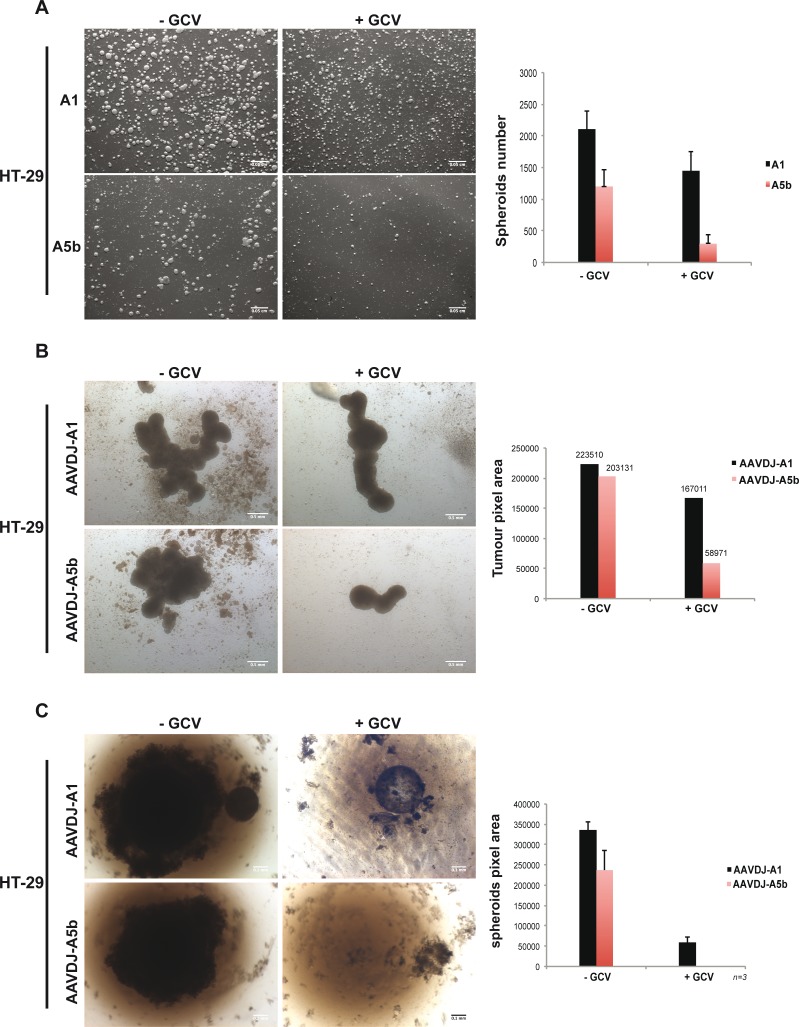
Targeting spheroid growth of HT-29 cells **A**. HT-29 cells were transfected twice as described above and 48 hours post transfection, 10^6^ cells were cultured in bacterial petri dishes, with or without GCV. One-week later, spheroids were imaged and counted. Scale bar; 50 μm. **B**. 10^5^ HT-29 cells were transferred onto agarose-coated 12-well plates and infected at 10^7^ MOI and GCV was added after 4 hours. Two weeks later spheroids were imaged and measured. Scale bar; 50 μm. **C**. Similar to MCF-7 cells above, HT-29 cells were grown as single spheroids in a 96 well plate with 2 cycles of infection and spheroids were imaged and measured after 1 month of growth. Scale bar; 50 μm.

In a parallel approach HT-29 cells were transfected and cultured in bacterial petri dishes where they formed multiple free-floating spheroids. After 7 days, the number and size of the spheroids derived from the A1 transfected cells was mildly reduced (30%) by GCV treatment, however this effect was much more potent in the spheroids derived from the A5b transfected cells with an 80% reduction in their number with respect to the absence of GCV (Figure 5A). HT-29 cells were also infected with the AAVs and grown on agarose-coated wells. After 14 days of culture, HT-29 cells formed large aggregated spheroid masses (Figure 5B). GCV treatment reduced spheroid size by 65% in A5b infected cells, compared to 10% in the A1 cells. Additionally HT-29 cells were also grown in agarose-coated 96 well plates under conditions were a single spheroid is produced per well. After 1 month, spheroid growth was completely abolished in the A5b infected cells (Figure 5C). As with the MCF-7 cells, after two serial AAV infections over a one month period, spheroid growth was completely inhibited only in cells infected with the A5b AAV and GCV treatment. Thus, spheroid growth of both of these cell lines can be completely abrogated using a combination of A5b-expressed HSV-TK and GCV.

## DISCUSSION

Here we describe a novel strategy for efficient and selective expression of the HSV-TK suicide gene in cancer cells based on their L1 ORF1/2p expression. We show that transfection with Alu-driven vectors or infection with the corresponding AAVs renders the tumour cells sensitive to efficient GCV mediated growth inhibition both as monolayers and spheroid cultures. We show that L1 ORF1/2 expression conferred GCV sensitivity to primary WI-38 and that this sensitivity was dependent on ORF2 RT-activity. These observations highlight the specificity of our strategy for selectively targeting cancer cells based on their reactivation of L1 expression and expression of functional ORF1/2 machinery.

Based on our data we suggest a new selective and non-toxic method for cancer treatment. Given the clinical experience of AAVs already used for human gene therapy [48], our strategy has potential to be adapted for use as an adjuvant to targeted inhibitor or immunotherapy for human cancers. Furthermore, the strategy could be further adapted by replacing the HSV-TK gene with other suicide genes or genes that mark cancer cells for elimination by the immune system.

## MATERIALS AND METHODS

### Vectors

pJM101L1.3 and pJM105L1.3 have been described elsewhere [45].

A2, A5, A5b, A12, A13a, A13b and A14 were designed in silico using SnapGene software (from GSL Biotech; available at snapgene.com) and were synthetized in Genscript^®^. Sequences and maps are available upon request.

### ORF1 antibody

Monoclonal anti-ORF1 was prepared as following; ORF1 protein fused at its N-terminus to 6XHistidine was expressed in *E.coli* and purified using Ni-NTA agarose (Qiagen). Mice were injected with purified recombinant ORF1 and monoclonal antibody production and screening were performed as previously described [49].

### Cell lines

MCF-7 cells were grown in DMEM (1g/l glucose) supplemented with 10% foetal calf serum, insulin 0.6 μg/ml and gentamicin 40 μg/ml. HT-29 cells were grown in DMEM (4,5g/l glucose) supplemented with 10% foetal calf serum and gentamicin 40 μg/ml. NCI-H1975 cells were grown in RPMI (2,5g/l glucose), HEPES 10mM supplemented with 10% foetal calf serum, Na Pyruvate 1mM and gentamicin 40 μg/ml. WI-38 cells (purchased from the ATCC repository in 2015) were grown in MEM (Life Technologies) supplemented with 10% foetal calf serum, AANE 0,1 mM and gentamicin 40 μg/ml. IMR-90 cells (purchased from the ATCC repository in 2015) were grown in MEM w/Earle's salts (Invitrogen) supplemented with 15% foetal calf serum, AANE 0,1 mM and gentamicin 40 μg/ml. MCF-7, HT-29 and NCI-H1975 cells were authentified by LGC July 2015. All cell lines are mycoplasma free (Venorgem mycoplasma PCR detection kit). HEK293T cells used for AAV production were grown in DMEM (4,5g/l Glutamax-I) supplemented with 10% foetal calf serum and gentamicin 40 μg/ml.

### Immunostaining

HEK293T cells were seeded on coverslips in a cell culture dish 96 hours after transfection with A5b or after co-transfection with HSV-TK and GFP expression vectors as positive controls. Cells were fixed for 10 minutes with 4% paraformaldehyde, washed with PBS and permeabilized with 1% Triton X-100 in PBS for 15 minutes, followed by washing with PBS and incubation with polyclonal anti-HSV-TK (1:2000 dilution) anti-serum at 4°C overnight. Next day, cells were washed with PBS and incubated with Alexa Fluor 555 conjugated-anti-rabbit IgG (1: 500 dilution; Invitrogen) for 1 hour, and nuclei stained with DAPI (Invitrogen, Life Technologies) for 2 minutes. The slides were embedded in Vectashield and fluorescence was captured by confocal microscopy (Leica Sp5 Laser Scanning Confocal Microscope, GE).

### AAV production

AAVDJ-A5b/A1 production was performed by the triple transfection method in HEK293T cells [50].

### Spheroid formation and infection

5×10^4^ MCF-7 cells were plated in a 24-well plate and immediately after plating, the cells were infected with AAVDJ-A5b or AAVDJ-A1 at a multiplicity of infection (MOI) of 10^7^ viral genomes per cell. At 48 hours post infection, the cells were trypsinized and grown as spheroids using a procedure adapted from [51]. 200μl of a cell suspension containing 7500 cells were transferred into one well of Agarose-coated 96-well plate (1.5% agarose in DMEM wt/vol), after 4 hours GCV was added and one week later, each spheroid was re-infected with 5×10^8^ viral particles.

The RS laboratory is supported by the Helmholtz Gesellschaft and INSERM Plan Cancer (épigénetique et cancer).

## SUPPLEMENTARY MATERIALS FIGURES


