# Supplementary material for: Selective elimination of long INterspersed element-1 expressing tumour cells by targeted expression of the HSV-TK suicide gene
Source: Oncotarget. 2017 Mar 8;8(24):38239–50. doi: 10.18632/oncotarget.16013 (PMC5503529; doi:10.18632/oncotarget.16013)
Supplement: Supplementary file 1 [file oncotarget-08-38239-s001.pdf]

Selective elimination of long INterspersed element-1 expressing tumour cells by targeted expression of the HSV-TK suicide gene

Supplementary Material

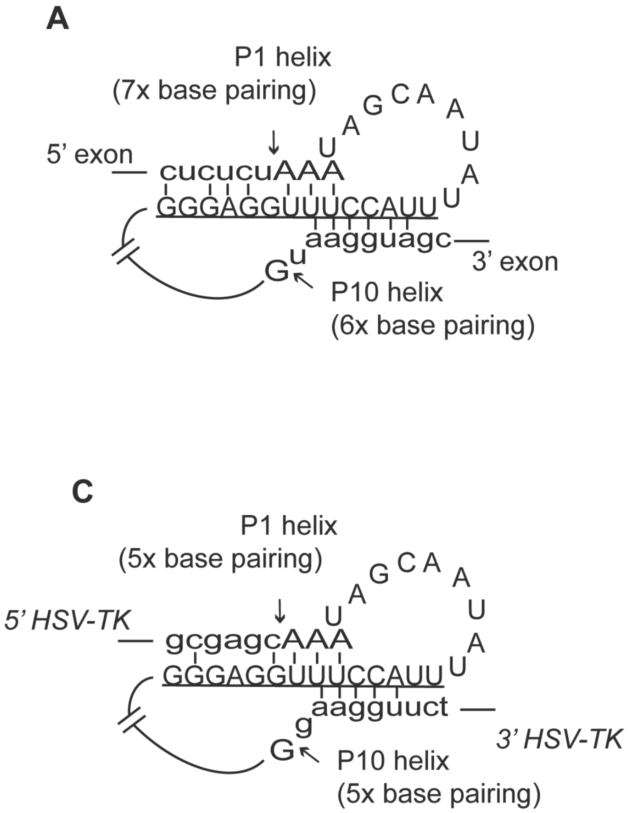

Supplementary Figure 1: **Schematic representation of the interactions involved in Tetrahymena intron self-splicing.** The pairing between the IGS and the 5' and 3' HSV-TK sequences are schematized. The IGS sequence is underlined and the bulk of the intron sequence is not represented. The arrows indicate the 5' and 3' splice sites, and the HSV-TK and Tet sequences are shown in lower and upper cases, respectively. The interaction of the IGS with the 5' and 3' exon sequences (P1 helix and P10 helix, respectively) are drawn simultaneously. **(A)** The wild type Tetrahymena with P1 containing 7 5' exon-IGS paired bases and P10 containing 6 3' exon-IGS paired bases. **(B)** Position 1 with 3 HSV-TK-IGS paired bases for both P1 and P10 (note that we replaced C in Tet wild type by A in red, to induce the third base pairing in P10). **(C)** Position 2 containing 5 HSV-TK-IGS paired bases. Adapted from Esnault C et al [38].
